# The US Military Commitment to Vaccine Development: A Century of Successes and Challenges

**DOI:** 10.3389/fimmu.2018.01397

**Published:** 2018-06-21

**Authors:** Silvia Ratto-Kim, In-Kyu Yoon, Robert M. Paris, Jean-Louis Excler, Jerome H. Kim, Robert J. O’Connell

**Affiliations:** ^1^International Vaccine Institute, Seoul, South Korea; ^2^GSK Vaccines, Rockville, MD, United States; ^3^Armed Forces Research Institute of Medical Sciences, Bangkok, Thailand

**Keywords:** vaccines, military medicine, army, development, history

## Abstract

The US military has been a leading proponent of vaccine development since its founding. General George Washington ordered the entire American army to be variolated against smallpox after recognizing the serious threat that it posed to military operations. He did this on the recommendation from Dr. John Morgan, the physician-in-chief of the American army, who wrote a treatise on variolation in 1776. Although cases of smallpox still occurred, they were far fewer than expected, and it is believed that the vaccination program contributed to victory in the War of Independence. Effective military force requires personnel who are healthy and combat ready for worldwide deployment. Given the geography of US military operations, military personnel should also be protected against diseases that are endemic in potential areas of conflict. For this reason, and unknown to many, the US military has strongly supported vaccine research and development. Four categories of communicable infectious diseases threaten military personnel: (1) diseases that spread easily in densely populated areas (respiratory and dysenteric diseases); (2) vector-borne diseases (disease carried by mosquitoes and other insects); (3) sexually transmitted diseases (hepatitis, HIV, and gonorrhea); and (4) diseases associated with biological warfare. For each category, the US military has supported research that has provided the basis for many of the vaccines available today. Although preventive measures and the development of drugs have provided some relief from the burden of malaria, dengue, and HIV, the US military continues to fund research and development of prophylactic vaccines that will contribute to force health protection and global health. In the past few years, newly recognized infections with Zika, severe acute respiratory syndrome, Middle East respiratory syndrome viruses have pushed the US military to fund research and fast track clinical trials to quickly and effectively develop vaccines for emerging diseases. With US military personnel present in every region of the globe, one of the most cost-effective ways to maintain military effectiveness is to develop vaccines against prioritized threats to military members’ health.

## Introduction

Infectious diseases occur worldwide (1, 2). It is therefore no surprise that militaries have throughout history been subject carriers, and vectors of infectious pathogens. Until World War II, the majority of deaths in military units engaged in combat were due to infectious diseases rather than direct combat injuries (3). Personnel lived in close quarters ate common prepared food and were exposed to poor sanitary conditions in the battlefield. Outcomes of military campaigns were often driven by the health conditions more than military preparedness (4). The threat of malaria was clear in the mind of Gen Douglas McArthur when, in 1943 he remarked to Dr. P. F. Russell: “this will be a long war if for every division I have facing the enemy I must count on a second division in hospital with malaria and a third division convalescing from this debilitating disease!” (5). Military epidemiologists were instrumental in the discovery of vector-borne diseases and mechanisms of transmission of many infectious diseases. Military doctors deployed with troops in the battlefield were able to study the environment and the diseases that affected the soldiers. Their experience informed vaccine development for many infectious diseases (4, 6, 7).

Warfare has changed in the quarter century since the end of the Cold War. Military operations have become smaller, faster, and asymmetric, with “complex operations other than war” (4). Military personnel may be stationed abroad for extended period of times with frequent contact with the local populations, vectors, and animals that increase the risk of exposure to diseases that are not a threat on US soil. For the same reason, monitoring emerging diseases and potential biowarfare pathogens has been an interest of the US military (8).

**Figure 1:**
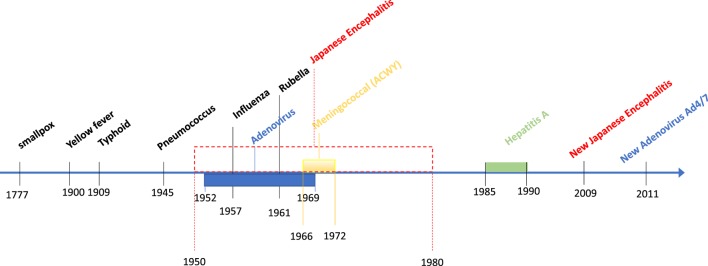
Timeline display of US military involvement in the research and development of major vaccines.

Developing safe and effective vaccines is a cost-effective solution to prevent infectious diseases and maintain healthy and combat-ready personnel. For this reason, the US Department of Defense (DoD) has funded vaccine research for several infectious diseases affecting people around the world (Figure 1). However, it is important to underline how vaccine manufacturing has become one of the most challenging processes because of its complexity and inherited uncertainties of vaccine research and development. The cost of developing safe and effective vaccines has greatly increased, and without innovation and continuous commitment, it will become an unsustainable and unobtainable goal for the US military.

The DoD conducts most of its endemic infectious diseases vaccine research at the Walter Reed Army Institute of Research and at the Naval Medical Research Center. Research and development of biowarfare countermeasures is conducted at the US Army Research Institute for Infectious Diseases at Fort Detrick, Maryland. These institutes operate overseas research units in multiple sites in Africa, Thailand, Georgia, Cambodia, Singapore, and Peru. A central mission of these institutes has been to study, design, and develop safe and effective vaccines that would protect US military personnel. Many young physicians started their careers in vaccinology in the US military and then moved to industry or academia where they continued to make important contributions to the field. Dr. Albert Sabin, the father of oral polio vaccine, was an Army major working in the Pacific Theater during World War II and contributed to the generation of the first Japanese encephalitis (JE) vaccine and to the epidemiology of dengue. In addition, the military recognized the benefit of being able to test vaccines in endemic areas where the epidemiology of the infectious disease of interest is well documented. Since the 1960s, the US Army maintained a collaborative effort with the Royal Thai Army (RTA) by establishing a South East Asia Treaty Organization Medical Research Laboratory in Bangkok, Thailand that became the Armed Force Research Institute of Medical Science (AFRIMS) in 1977. The collaborative effort between the Thai and US Army doctors at AFRIMS, the Ministry of Public Health (MoPH), and Thai academic institutions working in collaboration with the pharmaceutical industry has conducted vaccine efficacy trials for JE, hepatitis A (HepA), dengue, and HIV resulting in the licensure of vaccine for JE, HepA, and dengue (6, 9).

## The Early Years

### Smallpox

The first large-scale smallpox infection prevention campaign was conducted in 1777 by the Continental Army (10). General George Washington knew that the troops were vulnerable to smallpox and made the strategic decision to have soldiers variolated. This decision may have contributed to the defeat of the British in the Revolutionary War (1776–1783). The Army continued to vaccinate its recruits against smallpox until the early 1990s, 20 years after vaccination stopped in the civilian population and smallpox was considered eradicated (7). After the 2001 terrorist attack on the United States and the use of anthrax spores in a bioterrorism attack, smallpox once again was viewed as a potential threat to US military readiness (11). Smallpox is caused by an orthomyxovirus and poses a high epidemic risk (12, 13). Using smallpox as a biological weapon was unfortunately not new to warfare; sundries, blankets, and handkerchiefs were distributed to Native Americans in 1763 around Fort Pitt, Pennsylvania to decimate the local, native population (3). Restarting smallpox vaccination in a post-eradication world with a live vaccine was not without risk and serious adverse events (AEs) were reported (14, 15). After 2003, the military decided to vaccinate only individuals who were due to be deployed in “high risk” areas. New smallpox vaccine formulations associated with fewer AEs have been developed. Currently, the Strategic National Stockpile has three smallpox vaccines: ACAM2000^®^, the only licensed smallpox vaccine in the US; Aventis Pasteur Smallpox Vaccine (APSV); and Imvamune (Bavarian Nordic); the Center for Disease Control recommends routine vaccination only for specific populations at high risk of occupational exposure (16).

## Early Twentieth Century

### Yellow Fever

The US territorial expansion brought new challenges to the military. With the acquisition of Cuba after the Spanish-American war, US troops stationed on the island were decimated by yellow fever, a debilitating disease with an estimated 20% fatality rate (17). A young group of preventive medicine officers led by Major Walter Reed was able to contain the disease by identifying the route of transmission through mosquitoes and by implementing vector control measures, they were able to control the disease. Ultimately the etiology was identified as a filterable virus transmitted by *Aedes aegypti* mosquito and by the 1930s, a vaccine was developed and is currently still in use (18). Recent yellow fever epidemics in South America and Africa highlight the importance of yellow fever vaccination in endemic areas. The recent epidemics and a recent manufacturing problem of the only US licensed yellow fever vaccine YF-VAX^®^, produced by Sanofi Pasteur, have caused a vaccine shortage. By mid-2017, worldwide stockpiles were depleted and new vaccine manufacturing will not resume till mid-2018. This event has impacted the US Military and the general population (19). Both the Centers for Disease Control (CDC) and the DoD have developed contingency measures to counteract this threat by fractioning the vaccine dose as it was demonstrated that even lower doses were immunogenic (20, 21). An important message that emerges from this incident is that a closer monitoring of worldwide stockpiles of vaccines for preventable diseases remains key when there is only one (FDA approved) vaccine manufacturer.

### Typhoid

More US troops died in military training camps due to enteric fever caused by Gram negative bacillus *Salmonella enterica* serovar Typhi than died on the battlefield during the Spanish-American War. The same scenario unfolded during the Anglo-Boer War where 8,225 British troops died of typhoid compared to 7,582 of wounds (7, 18). A British pathologist, Sir Almroth Wright was the first to develop a typhoid vaccine at the Army Medical School, Netley, England. He pioneered a vaccine preparation method that involved heat inactivation of bacilli taken from an infected patient. After his success, Major Frederick Russell of the US Army modified the vaccine formulation and after establishing the safety and efficacy of the vaccine, typhoid immunization became a requirement for all US troops after 1911. Consequently, the US Army had the lowest typhoid fever incidence of any of the major combatants in World War I. With improvement in sanitation systems enteric fever due to *S*. Typhi has become rare in the developed world but in low- and middle-income countries where clean water and sanitation are still a challenge, it infects 20 million people and kills over 100,000 every year (22).

### Pneumococcus

*Streptococcus pneumoniae* was discovered by Major George Sternberg in 1881, the same year as Louis Pasteur’s seminal discovery (23). Upper respiratory diseases were a major problem for troops, which spurred the Army to develop a vaccine against pneumococcal pneumonia. By 1930, polyvalent pneumococcal vaccines were tested at different sites, but the final successful clinical trial was performed in Sioux Falls in 1944–1945 at the Army Air Force Technical School where a high incidence of pneumococcal pneumonia was found (6). The vaccine did not have a great impact because the greater availability of penicillin lessened the need for a vaccine in healthy young adults. Subsequently, multivalent pneumococcal vaccines were introduced for the elderly (24) and ultimately the development of conjugated 10 or 13 valent formulations have drastically reduced the rate of invasive pneumococcal disease in infants, saving millions of lives since their introduction in the early 2000s (24–26).

## Mid Twentieth Century

### Influenza

During World War I, the pandemic of Spanish influenza had a devastating impact on the US military, claiming the lives of over 43,000 sailors and soldiers. The fast spread of the disease was aided by transport of troops across the oceans where close quartering contributed to the spread of the virus. The Armed Forces Epidemiological Board (AFEB) which evolved from previous Military Infectious Diseases Boards that were commissioned to study the epidemiology of influenza and other highly occurring infectious diseases was led by Thomas Francis Jr., the first scientist to isolate the influenza virus from an infected human (18). The work of AFEB was instrumental in the preparation of the first whole-inactivated virus vaccine tested in hospitalized inmates, military recruits, and college students. The first inactivate strain was type A influenza and sooner after the first vaccination season the inactivated B virus strain was added, and the first bivalent influenza vaccine was used to vaccinate troops in 1945. Data on flu vaccine efficacy showed that new strains were appearing in circulation each season leading to changes in vaccine virus composition to match the circulating influenza strains (18, 27). The US military has continued to study the influenza virus and the quest for a more effective vaccine, and epidemiologists have made recommendations on the composition of the yearly flu vaccine through the years (18, 27–31).

### Adenovirus Type 4 and 7

As military scientists were investigating influenza infections at Fort Leonard Wood in 1952–1953, they realized that another virus with similar symptomatology was causing an acute respiratory disease (ARD) in recruiting and training camps. Adenovirus 4 and 7 was isolated from these soldiers. A formalin-inactivated adenovirus type 4 and 7 vaccine was introduced in 1956 and was soon replaced by an oral formulation that was 50% effective in reducing hospitalization caused by ARD (18). Wyeth Pharmaceuticals provided the vaccine for the US military until 1996 when production was halted. As a result of stopping vaccinations, the rate of adenovirus infections at the recruiting training centers increased dramatically in the following years (18). The cost of hospitalization and toll on military personnel health and readiness was a deciding factor for the US Army to enter into contract with Barr Pharmaceuticals in 2001 to resume production of the Ad4 and Ad7 vaccines (18). After clinical trials showed high seroconversion rate and safety profile, the Ad4 and Ad7 vaccines were approved by the US FDA and vaccination resumed in 2011 at all military recruiting centers. A follow-up study showed that after 2 years of vaccination, a 100-fold reduction in disease burden was observed in the recruit population (32).

### Rubella

The rubella virus was first isolated by three military scientists (Captain Paul Parkman, Captain Malcom Artenstein, and Lieutenant Colonel Edward Buesher) from a recruit hospitalized at Fort Dix during an adenovirus outbreak in 1961 (7). The isolation of the virus prompted the development of the first rubella live-attenuated vaccine, available for the general population in 1969 (18, 33, 34). Several improvements were then brought to the original vaccine (strains, cell substrate) (34). Since then, rubella cases have steadily declined through the years and the occurrence of congenital rubella syndrome (CRS) has been drastically reduced. CRS can affect virtually every organ and the severity increases if the infection occurred early in gestation (34). The measles-mumps-rubella combination vaccine was critical to the reduction of the devastating impact of CRS (35).

### Japanese Encephalitis

The Japanese encephalitis virus (JEV) was first isolated in 1935 from the brain of a patient who died of encephalitis in Japan. In early 1940, Major Albert Sabin was assigned the task of developing a vaccine against JEV. He and his team produced a formalin-inactivated vaccine from JEV-infected mouse brain that was administered to more than 250,000 military personnel during World War II. With the continued presence of US military personnel in Asia in the 1950s and during the Korean War, it became apparent that the vaccine was not efficacious enough, so vaccination was halted and research on a better vaccine commenced. In the mid 1980s, the US Army conducted the pivotal study in Thailand that led to the US FDA approval of a JEV vaccine (JE-VAX), a new whole virus formalin-inactivated vaccine (7, 18). This virus was still produced from mouse-infected brains and although it was deemed safe, concerns remained and the two pharmaceutical companies that produced and distributed JE-VAX (BIKEN and Sanofi Pasteur, respectively) stopped production in 2005. Because of the continuous threat of JE together with the need for military personnel to be station throughout Asia, the US military remained engaged in the search for a second-generation JEV vaccine preparation. Promising results with a new JEV vaccine formulation developed by Intercell AG and tested in phase I by WRAIR scientists led to the full development and approval of IXIARO (36–38). IXIARO is a JEV attenuated SA-14-14-2 strain grown in Vero cells, this vaccine was approved for use in children in 2013, and it is registered in several endemic countries. IMOJEV^®^ (JE-CV and previously known as ChimeriVax™-JE) is a novel recombinant chimeric virus vaccine developed by Sanofi Pasteur using the yellow fever virus (YFV) vaccine vector YF-17D, replacing the cDNA encoding the envelope proteins of YFV with that of the attenuated JEV strain SA14-14-2. IMOJEV^®^ single dose was found to be safe, highly immunogenic, and capable of inducing long-lasting immunity in both preclinical and clinical trials. It has been tested in the US military personnel (39).

### Meningococcal Vaccine

Meningococcal disease, caused by the bacterium *Neisseria meningitis*, is associated with outbreaks among personnel in highly confined settings such as military training camps and university campuses. Outbreaks were commonly reported in military recruits since the nineteenth century, but during the Vietnam War (1964–1971), an epidemic of serogroup B and C meningococcal meningitis among US army recruits resulted in the closing of Fort Ord in California. The death rate from the epidemics during this period was similar to those due to malaria. The concurrent surge of antimicrobial resistance pushed the US military to accelerate vaccine research. The human immunological response to the bacterium served as the basis for the first polysaccharide vaccine against serogroups A and C. In 1969, four major papers were published by US Army researchers that defined the assay for bactericidal antibody using human complement that was accepted as a correlate of protection in humans and served as the basis of licensure for all existing meningococcal vaccines (40–43).

Phases I–III were conducted by the US military leading to a licensed vaccine to serogroup A followed by a combined serogroup A/C vaccine in 1970 and 1978, respectively (18). Meningococcal serotype B vaccine was harder to develop because its antigens have homology with human neuronal proteins. Although the US military was not involved with the development of this vaccine, it is worth noting that through reverse vaccinology, a method of vaccine design that starts with the prediction of antigens from the genome sequence of a meningococcal B strain (MenB), two new products are now available (44, 45).

### Hepatitis A

Hepatitis A (Hep A) virus causes hepatitis epidemics in military personnel deployed in areas with poor sanitary conditions. The epidemics rarely caused death, but servicemen would develop jaundice, be indisposed, hospitalized, and unable to fight. The US military doctors first demonstrated that immunoglobulin could prevent or attenuate Hep A disease, however, protection was temporary and needed continuous re-injections that were unfeasible for long deployments in endemic areas. Therefore, WRAIR scientists sought to develop an effective vaccine. They discovered the best method of culture of Hep A virus and established in animal models that one serotype could protect against strains from other endemic areas. In 1986, they produced the first formalin-inactivated vaccine tested in humans. The phase III trial of Hep A vaccine commenced in 1991 in Thailand through a collaboration of the Thai MoPH and Smith Kline Beecham Biologics (now GSK). It involved 20,000 children vaccinated with Hep A vaccine and 20,000 with Hep B vaccine as control. The success of this trial brought the Hep A vaccine (HAVRIX) to licensure in 1995 (18).

## Current Challenges

Several other debilitating infectious diseases represent a serious public health threat, in particular for the military personnel and for which a preventive vaccine is not yet available.

### Malaria

Malaria, a mosquito-borne infectious disease, is derived from the Italian word that comes from the contracted form of *mala aria* or “bad air,” referring to the “intermittent fevers that affected people living near marshy districts and attributed to the unwholesome airs that were produced by the stagnant waters.” In 1775, the first US Continental Congress appropriated $300 for the first medical acquisition of “Peruvian bark” for the treatment of fever. This was prior to the discovery of the malaria parasite, but it was well recognized at the time that the bark of the cinchona tree, from which quinine is extracted, was effective in treating malarial fever (46). It was not until the 1880s when the French army surgeon, Charles Louis Alphonse Laveran first noticed the appearance of parasites (*Plasmodium* spp.) in the blood of a patient suffering from malaria in Algeria. In 1900, Col. William Crawford Gorgas with other Army colleagues recognized the importance of vector-borne mosquito transmission of infectious diseases to humans and implemented one of the most effective vector control programs in Panama in 1904. Within 3 years the incidence of malaria was reduced from 800 cases/1,000 workers to just 16/1,000 (47).

Until World War II, the military strategy against malaria remained primarily vector control. In 1943, the introduction of dichlorodiphenyltrichloroethane (DDT) greatly aided those efforts. During World War II, quinine, used for both treatment and chemoprophylaxis, was in short supply for Allied troops because the majority of cinchona plantations were located in Java (the Dutch East Indies) which was controlled by the Japanese. It became clear that new drugs, and a vaccine, were needed to maintain effective force protection. A malaria drug development program was started that included academic, government, industry, and military partners in an unprecedented effort to discover new antimalarial drugs. This highly classified program resulted in the discovery of chloroquine and primaquine for the treatment and prophylaxis of both falciparum and vivax malaria (48). After World War II, the US DoD remained a leading investor in malaria drug and vaccine development, which was reinvigorated by the Vietnam war and the spread of chloroquine resistance (49). It was during this time that WRAIR emerged as a lead developer in new antimalarial drugs as well as malaria vaccines (47, 50).

Due to the complexity of the malaria parasite life cycle in humans and mosquitoes, that includes asexual and sexual stages, it has been difficult to develop an effective vaccine. Early clinical experiments done by the University of Maryland and the WRAIR showed that irradiated, infected mosquitoes could transfer attenuated *P. falciparum or P. vivax* sporozoites through multiple infected mosquito feedings. The immune response generated conferred subsequent protection against wild-type falciparum malaria in controlled human malaria infection model (CHMI) (51, 52). These early studies, although crude, demonstrated that a vaccine against malaria was possible. A biopharmaceutical company (Sanaria) has devised a method to purify malaria sporozoites (the infective stage of the parasite) from irradiated, aseptic mosquitoes and store the irradiated sporozoites in a stable, frozen formulation. Irradiated sporozoites (referred to as PfSPZ) are thawed and administered intravenously. The PfSPZ vaccine has been tested in phase I/II clinical trials and demonstrated to protect against clinical malaria using a well-established CHMI model (53, 54). Though recent field trial results were mixed (55), this method of vaccination is being pursued, though production and scale up remain as significant hurdles (56). Another approach spearheaded by WRAIR scientists has been the use of a recombinant protein approach based on the circumsporozoite protein (CSP) of the pre-erythrocytic (sporozoite) stage of the malaria parasite. CSP was one of the first surface-expressed, GPI-anchored proteins cloned (57) from *Plasmodia* and was shown to be a key target for protective immunity-induced by irradiated sporozoites in animal models as well as in clinical malaria (58, 59). This strategy was undertaken in collaboration with Smith Kline Beecham (subsequently GSK), which resulted in the initial testing of the RTS,S malaria vaccine candidate in combination with several novel adjuvants by US Army investigators (60). RTS,S consists of a single fusion protein composed of the CSP central *R*epeat region and *T cell* epitopes with hepatitis B *S*urface antigen. This is co-expressed with free hepatitis B surface antigen in yeast cells, resulting in self-assembling viral like particles (61). Initial promising results led to the clinical development of RTS,S adjuvanted with AS01_E_ through a pivotal phase III efficacy trial. The vaccine was given a positive scientific opinion after review by the European Medicines Agency for use outside the European Union (62–64). Phase III testing in children aged 5–17 months, showed efficacy of 51.3% against all episode of clinical malaria over 12 months at all site tested. Efficacy was lower at 18 months and was further reduced after 3 years of follow-up. A fourth vaccination seems to increase slightly efficacy overall at 32 months. WHO recommended this vaccine to be tested in small pilot studies to understand if the data can be replicated in the normal health care delivery system (65). However, this level of efficacy would not be considered sufficient for force health protection [as compared to traditional chemoprophylaxis which is ~90% effective with good adherence (66)]. These observations led to renewed interest in further assessment of the dose and schedule of RTS,S based on the initial results of et al. in 1997 where a regimen of 0, 1, and 7 months with a fractional third dose (1/5th of the first two doses) resulted in six of seven participants (87%) protected from controlled human malaria infection (60). A subsequent phase IIa trial, conducted in 2013, replicated these results, protecting 26 of 30 subjects (87%) using the CHMI model (67), suggesting that further improvements to the efficacy of this approach are feasible and warrant further clinical development (68).

### Dengue

Dengue fever is a mosquito-borne disease caused by one of four serotypes of dengue virus (DENV), a flavivirus transmitted by *Aedes aegypti*. DENV causes a febrile illness that can occasionally be fatal, especially if managed poorly. Dengue is more likely to be severe upon infection with a second serotype different from an initial infecting serotype, and can be associated with plasma leakage, severe bleeding, respiratory distress, and organ impairment. An estimated 50–100 million annual symptomatic dengue infections are associated with 500,000 cases of severe dengue and about 20,000 deaths. Overall, the mortality rate is low (<1%) if managed properly. The quest for an effective dengue vaccine started more than 50 years ago, but an effective and safe vaccine has proved elusive. US military personnel have dealt with dengue since the beginning of the twentieth century during the Spanish-American War. In 1906, a dengue epidemic affected troops stationed at Fort McKinley, Manila, Philippines, and the Army Tropical Disease Board made the study of DENV a priority. During World War II in the South Pacific, the rapid expansion of troops and bases permitted DENV to spread from island to island on planes and ships used to supply bases. By 1944, most islands in the South Pacific had identified cases of dengue, and it was estimated that dengue in Melanesia and neighboring islands caused more than 80,000 sick days and infection rates of 12% among US troops (69). During and after World War II, Major Albert Sabin isolated DENV serotypes 1 and 2 from Hawaii and New Guinea, and William M. Hammon identified DENV serotypes 3 and 4 from the Philippines as the cause of hemorrhagic fever (7, 18, 69). There is no current anti-viral medication available for DENV infection. Monitoring of dengue cases and judicious fluid replacement have reduced the mortality rate to less than 1% (70, 71). Given the high attack rates and substantial burden of symptomatic illness, the military has focused on the development of a safe and effective dengue vaccine.

### Dengue Vaccine Development: Lessons Learned and Current Challenges

Dengue vaccine has been in development for over 50 years and has presented a challenge because of the unique characteristic of the immune responses to the four virus serotypes, lack of immune correlates of protection, and lack of suitable animal models (72). A dengue vaccine sponsored by Sanofi Pasteur has been licensed in multiple dengue endemic countries, and several candidate vaccines are at various stages of development (73). Although the US military has maintained interest in several different candidate vaccine to prevent dengue, Sanofi Pasteur in 2010 initiated the first phase 2b proof of concept trial using their CYD tetravalent dengue vaccine (CYD-TDV). The trial was conducted in Thailand among 4,000 children aged 4–11 years. CYD-TDV is composed of four chimeric live-attenuated viruses (CYD1–4) based on a yellow fever vaccine backbone (YF 17D) with structural DENV proteins (74). Preclinical and clinical studies have shown that a three-dose vaccination regimen induced balanced immune responses to all four serotypes, and pre-existing flavivirus infection seemed to induce a more rapid immune response with no increase in vaccine-derived viremia. Unfortunately, this tetravalent vaccine did not provide equal protection against all four dengue serotypes, with especially low efficacy against DENV-2 (75). Subsequent multi-country phase III trials in Asia and Latin America in 2- to 16-year-old children showed good efficacy against DENV-3 and 4, moderate efficacy against DENV-1 and marginal efficacy against DENV-2. Notably, an increase in relative risk of severe dengue in vaccine recipients aged 2–5 years during the third year of the Asian phase III trial (76, 77) led to the age indication of children 9 years of age and above. CYD-TDV (trademarked as Dengvaxia^®^) eventually received licensure in 20 dengue endemic countries but has not yet been approved in the US. Based on an assessment of potential overall public health benefit, WHO recommended in July 2016 that vaccination could be carried out in highly endemic areas (>70% dengue seropositive rates) (65, 78). However, in November 2017 Sanofi, who continued to monitor safety of their vaccine, announced the results from a new laboratory test that could infer dengue serostatus prior to vaccination in subjects from their phase III trials and found that seronegative persons of any age (including those >9 years) who receive Dengvaxia^®^ had a higher risk of severe dengue. On April 19, 2018, the WHO Strategic Advisory Group of Experts on Immunization revised their recommendations, emphasizing individual testing before vaccinating in order to minimize the likelihood of seronegative individuals receiving Dengvaxia^®^.^1^

The US army initially focused on tetravalent live-attenuated vaccine candidates, entering into a partnership with GSK in early 2000. A tetravalent live-attenuated dengue vaccine candidate was eventually evaluated in a phase II clinical trial in Puerto Rico (79). Subsequently, a purified, inactivated whole virus approach was pursued with GSK (80, 81), leveraging GSK’s proprietary adjuvants to try to elicit more durable tetravalent immune responses (72). US Navy has primarily pursued a DNA vaccine approach, evaluating a tetravalent DNA vaccine candidate in a phase I trial (82). Takeda has developed a tetravalent recombinant attenuated vaccine, TDV, based on a common, molecularly cloned DENV type 2 called DENVax-2. Serotypes 1, 3, and 4 are represented in the vaccine by substituting prM and E genes of DENVax-2 with those of their respective serotypes. TDV has undergone phase II trials and is currently in the midst of a large multi-country phase III efficacy trial in Asia and Latin America (NCT02747927), that includes a US Army site in the Philippines. US NIH developed their own tetravalent recombinant attenuated vaccine candidates, TV003/TV005, and has sponsored the candidate through phase I and II trials, including a trial in Thailand with the US Army. US NIH provided licenses to various manufacturers for ongoing product development, including Butantan, Vabiotech, Panacea, Serum Institute of India, Indian Immunologicals Inc., Medigen, and Merck. Butantan is currently conducting a large phase III efficacy trial of the vaccine in Brazil (NCT02406729).

### Human Immunodeficiency Virus

HIV poses a significant and persistent threat in terms of readiness and force protection and may act as a war-starter by affecting the stability and security of nation-states. In 2001, the Armed Forces Epidemiology Board identified HIV as a disease of military importance; the 2001 DoD Report on Biological Warfare Defense Vaccine Research and Development identified HIV as the fourth greatest infectious disease threat to DoD forces. Department of Army Headquarters designated HIV vaccine development as an Army Technology Objective, a status reserved for the highest priority science and technology efforts.^2^

HIV military relevance has been recognized from the very beginning of the pandemic. In 1985, the US military recognized the emerging HIV-1 epidemic as a new threat to US and allied forces worldwide. The United States Congress mandated the establishment of the US Military HIV Research Program (MHRP) to develop effective preventive measures to include prevention education, vaccine development and implementation of novel anti-viral therapies, and clinical management tools for the US DoD and Allied Forces (83). Much of the early HIV vaccine development in the Army focused on developing a vaccine against strains (subtype B and CRF01_AE) found in Thailand, because significant rates of HIV infection from heterosexual spread were found in RTA recruits from Northern Thailand and because of the strong and successful partnership between the US Army through AFRIMS and the RTA (84). The well-developed health surveillance system developed by the Thai MoPH together with the RTA was instrumental in the early collection of samples that allowed the scientists at AFRIMS and WRAIR to show that the majority of HIV-1 circulating in Thailand was a recombinant form (CRF01_AE) together with the already known North America B serotype (85). Thailand’s strong public health infrastructure and the early adoption of standardized HIV testing among the RTA recruits gave detailed information on the prevalence of HIV infection among the different geographical regions and the general Thai population. The collection of data further documented that aggressive education and behavioral interventions that were implemented by the government and non-for-profit organizations were effective in reversing the epidemic (86). The Thai AIDS Vaccine Evaluation Group that was established early on as a way to bring together the RTA, US Army already working together at AFRIMS and the major university research centers in Thailand (84). The first set of phase I trials tested recombinant envelope proteins alone or in combination that were derived from both circulating HIV strains (Table 1) (87, 88). The vaccinations were well tolerated and induced strong antibody responses. The addition of a canarypox prime (ALVAC-HIV) improved cellular immune responses. ALVAC-HIV was tested in phase I/II trials in the US and demonstrated good cellular immunogenicity but poor antibody responses (89–91). Prime/boost combinations were tested in the US and Thailand (92, 93) and by early 2000 Sanofi Pasteur and VaxGen entered an agreement to test their products in a prime-boost phase III trial (RV144). This HIV efficacy trial involved 16,402 community risk Thai individuals recruited in Rayong and Chonburi provinces through a partnership between the US Army, NIH, RTA, MoPH and Mahidol University. RV144 was the first and remains the only HIV efficacy trial to show protection, with vaccine efficacy of 31% at 42 months (94).

**Table 1:** HIV phase I/II/III tested by the US Army.

Vaccine	Trial (# of participants)	Company
Gp120SF2(B)/MF59 (87)	Phase I (*n* = 52)	Chiron
Gp120SF2(B)/MF59 + gp120CM235/MF59 (88)	Phase II (*n* = 370)	Chiron
ALVAC-HIV (vCP1521) prime + oligomeric gp160 (92TH023/LAI-DID) or ALVAC-HIV (vCP1521) prime + bivalent gp120 (CM235/SF2) (93)	Phase I/II *n* = 130	Sanofi Pasteur and Novartis Vaccine and Diagnostics
ALVAC-HIV (vCP1521) prime + AIDSVAX B/E (92)	Phase I/II *n* = 122	Sanofi Pasteur and VaxGen
ALVAC-HIV (vCP1521) prime + AIDSVAX B/E (94)	Phase III *n* = 16,402	Sanofi Pasteur and VaxGen
MVA CMDR (CRF01_AE) (95)	Phase I *n* = 48	LVD/NIAID/WRAIR/MHRP
PENNVAX-G DNA + MVA-CMDR (96)	Phase I *n* = 88	Innovio Pharmaceutical + LVD/NIAID/WRAIR/MHRP

RV144 also established a correlate (biomarker) associated with decreased risk of HIV infection (antibody to the HIV gp120 V1V2 region) (97). The study led to a series of additional insights regarding potential correlates of risk (98) and had greatly informed the ongoing ALVAC + g120 efficacy trial in the Republic of S. Africa. The US MHRP continues to invest in prime-boost strategies with a variety of immunogens (95, 96) (Table 1). The US military has maintained a strong support for HIV vaccine research and development and continuous monitoring of the epidemic.

### Enteric Diseases

Although personal hygiene, sanitation measures, and antibiotics have greatly improved conditions in military training camps, installations, and combat field sites, enteritis continues to plague military forces during deployments (99, 100). A few studies have tried to understand the days-work lost during military deployment to justify the founding for enteric vaccines (101, 102). Enteric diseases *per se* are not life threatening and although the burden of time lost for soldiers may not be substantial it is imperative to consider the effect that these infections may have on the population at large (101). Vaccines against enteric diseases may benefit deployed soldiers and their families in high-risk areas and there is secondary benefit for leisure travelers as well as populations living in low- and middle-income countries where hundreds of thousands die annually of diarrheal diseases. Besides acute illness, diarrheal diseases may cause chronic debilitating conditions like Guillain–Barre syndrome after infection with *Campylobacter* and reactive arthritis in 5% of individuals after *Shigella* and *Campylobacter* infection. Post-infection irritable bowel syndrome is now recognized as a sequela of infectious diarrhea and occurs in approximately 10% of individuals post-gastroenteritis. These chronic conditions may decrease work hours, quality of life, and increase the health cost burden to society (101).

The US Army has invested heavily on the development of enteric vaccines focusing primarily on three pathogens: Enterotoxigenic *E. coli* (ETEC), *Shigella*, and *Campylobacter* as they are considered the most important threat to troops worldwide. The US military has many different vaccines in the pipeline at various stages of development. Briefly, an attenuated *Shigella* vaccine (WRSS1) is in a phase IIb clinical trial, and one *Shigella* inactivated vaccine is in phase I clinical testing. Also, subunit vaccine like for *Shigella flexneri* 2a (Invaplex) and the bioconjugate Flexin2a are in preclinical development. Subunit protein made of fimbrial tip adhesin of ETEC CF proteins have been also tested by the US Naval Medical Center in phase 2 clinical trials (103).

### Rickettsial Diseases and Scrub Typhus

Human rickettsial diseases were grouped as “typhus fever” as they shared common symptoms. As diagnostics improved, it was discovered that the rickettial disease could be divided into three distinct groups: (1) tick typhus group (Rocky Mountain spotted fever as an example), (2) typhus group (louse born or epidemic typhus), and (3) scrub typhus group. The Rickettsiae are proteobacteria and can be transmitted by mites, fleas, flies, ticks, and lice. Mortality rates vary by species but can be high during period of war, famine and social disruption or because of underdiagnoses. The first clear account of Typhus fever occurred during the military siege of Granada in 1489 where 17,000 deaths were reported in the Spanish Army (104). Typhus devastated Napoleon’s troops during the invasion (and retreat) of Russia in 1812. Rickettsial diseases were present through World War I and II but since the etiology was discovered and troop’s hygiene improved, the incident cases also diminished. The use of the insecticide DDT, various chemical repellents, and the discovery of antibiotics collectively reduced the burden of rickettsial diseases. In addition, military troops on both fronts had access to various effective vaccines and most of the casualties were among the civilian populations (104).

The only reported rickettsial disease cases and deaths during World War II were from Scrub typhus infection (*Orientia tsutsugamushi*) in the Asia-Pacific region. Military scientists from WRAIR and University of Maryland discovered in 1948 that the antibiotic chloromycetin was effective against Scrub typhus but eventually resistance emerged (104). During the Vietnam War, Fever of Unknown Origin was caused mainly by Scrub typhus and had a co-infection incidence of 6% with Malaria. Until World War II, Scrub typhus was considered a sub-tropical disease, but it became apparent during the following year when US troops were stationed in Japan and Korea that seasonal Scrub typhus was present in these areas as well.

### New Challenges

#### Chikungunya Resurging

The Chikungunya virus is transmitted by *Aedes* mosquitoes and was first isolated in 1953 in Tanzania. Symptoms of Chikungunya infections include abrupt onset of fever with acute arthralgia and arthritis that can last for a very long time. In 1962, the US Army isolated a strain of Chikungunya from an individual in Thailand and started the development of an attenuated vaccine. A chikungunya vaccine was eventually developed by a partnership between the Salk Institute-Government Service Division under contract with the DoD in 1984. At the time, a review of the funding priorities for potential disruptive diseases for military operations, ranked Chikungunya low on the scale of threats to the military and as consequence the project was halted. In 2005, a resurgence of Chikungunya infection was observed in Kenya and Reunion Island where tens of thousands of individuals were infected and over 200 fatalities were reported (105). Representatives of the French Ministry of Health contacted the US Secretary of Health and Human Services as they were aware of the US Army’s previous work and several pharmaceutical companies also expressed interest (105). Currently, formulations of live-attenuated Chikungunya vaccine similar to the product shelved in the mid 1990 have been tested in a phase II trial, together with other similar strains that were obtained from the US Army laboratories (106). Hopefully the vaccine will find funding through licensure as it is considered a re-emerging infection in low- to middle-income countries.

#### Zika Virus (ZIKV)

Even though ZIKV has been known since 1947, its spread and consequent illness reached pandemic proportion only in 2013. It is currently present in more than 80 countries and causing millions of infections yearly (107). ZIKV is transmitted by the Aedes mosquito, which is ubiquitous and favors urban areas as breeding ground. It is transmitted from mother to fetuses, *via* sexual intercourse and possibly *via* transfusion and organ transplantation (107). The ability of ZIKV to cause both dengue-like febrile symptoms and neurological conditions (Guillain–Barre syndrome and encephalitis) and to cause marked teratogenicity, makes it a formidable foe for public heath control effort and consequently for military operations. Early reports of infection with ZIKV were out of Africa but the disease presentation was confounded by co-infection with other diseases. Diagnosis was hampered by cross-reactivity with closely related flaviviruses. It is safe to assume that infection with ZIKV had probably occurred but was unrecognized or misdiagnosed as dengue or JE and never reached epidemic proportions (108). The first epidemic was reported in Yap, Federated States of Micronesia, in 2007, followed by an outbreak in the French Polynesia in 2013. There was subsequent spread throughout the South Pacific. In 2015, a major epidemic of neurological disease in infants occurred in Brazil and rapidly spread through the Americas. Singapore and Vietnam were the sites of two outbreaks, and there was widespread infection in Thailand in 2017 (108). This emerging disease was declared a Public Health Emergency of International Concern by WHO in 2016. More than 40 candidate vaccines are in preclinical stages and 7 are currently being tested in phase I throughout the world. The US military research group at WRAIR in collaboration with the Beth Israel Deaconess Medical Center is testing a ZIKV purified inactivated virus based on their previous experience with JEV vaccines (109). Currently, new Zika infection rates have dramatically plunged in South America, possibly due to “herd immunity.” Nevertheless, the quest for an effective vaccine must remain at the forefront to combat this debilitating disease (110).

#### Hanta Virus

Although only discovered in 1993, Hanta virus can infect humans through exposure to aerosolized rodent’s excreta; infection causes hemorrhagic fever with renal syndrome (HFRS, old world rodents) or hemorrhagic fever with pulmonary syndrome (HFP, new world rodents). Most infections occur in China (111). The US military has justified the need for a vaccine by outlining the risk of exposure that troops could face in natural disasters or wars (particularly on the Korean peninsula), where destruction of human environments and stress on population may increase exposure to Hanta virus. A clear example of this risk was brought to light during the conflict in Bosnia Herzegovina; a serosurvey for Hanta virus among soldiers showed elevated rate of exposure (16.1%) compare to the population living in the endemic areas (6.2%) (111).

Because China and Korea have had the greatest number of HFRS, both countries have developed a brain-derived inactivated HFRS vaccine which, together with public health education, have reduced but not contained cases of HFRS. This vaccine is not licensed outside Asia and does not cross-react with the Hanta serotypes circulating in Europe.

The US Army tested an HFRS vaccinia vectored vaccine, but it was poorly immunogenic in humans who were already expose to vaccinia (112). A DNA-based vaccine was subsequently developed. New vaccines, which carry Hantaan and/or Puumala M segments to induce broader immunity, were tested in a phase I clinical trials in three cohorts and showed promising results (113).

## Bioterrorism

Not only are endemic diseases of concern for the military, so are potential exposures to agents deliberately introduced into the environment through biological warfare (BW) or bioterrorism (114).

Although President Richard Nixon terminated the offensive biological weapons program in 1969 and 1970 by executive order, research efforts in biowarfare countermeasures continue (115). During Operation Desert Shield before the Persian Gulf War and after the 9/11 events and the anthrax attacks on US institutions, it has become evident that BW remains a potential threat to US soldiers.

Bioweapon threats could include the deliberate release by attackers of an agent that causes one or more of a variety of different diseases. Public health authorities have developed a system to prioritize biological agents according to their risk to national security. Category A agents are the highest priority, and these are disease agents that pose the greatest risk to national security because they can be transmitted from person to person and/or result in high mortality, and/or have high potential to cause social disruption. These are anthrax, botulism (*via* botulinum toxin, which is not passable from person to person), plague, smallpox, tularemia, and a collection of viruses that cause hemorrhagic fevers, such as Ebola, Marburg, Lassa, and Machupo. These disease agents exist in nature (with the exception of smallpox) and could be manipulated to make them more dangerous. Category B agents are moderately easy to disseminate and result in low mortality. These include brucellosis, glanders, Q fever, ricin toxin, typhus fever, and other agents. Category C agents include emerging disease agents that could be engineered for mass dissemination in the future, such as Nipah virus (CDC index of possible threats).

The use of effective vaccines would likely protect lives and limit disease spread in a biological weapons emergency. Licensed vaccines are currently available for a few threats, such as anthrax and smallpox, and research is underway to develop and produce vaccines for other threats, such as tularemia, Ebola virus, and Marburg virus. Many bioweapon disease threats, however, lack a corresponding vaccine, and for those that do, significant challenges exist to their successful use in an emergency situation.^3^

The DoD Joint Vaccine Acquisition Program has several experimental vaccines in development (Table 2). These vaccines will be further developed and tested with the intent of obtaining products licensed by the US Food and Drug Administration (12, 116, 117).

**Table 2:** Vaccines for bioterrorism.

Licensed vaccines	Vaccines in R&D
Smallpox vaccine	Vaccinia (cell culture)
Anthrax	Botulinum toxoids
Plague	Tularemia
	Ricin toxin
	Q fever
	VEE, EEE, WEE

*VEE, Venezuelan equine encephalitis; EEE, Eastern equine encephalitis; WEE, Western equine encephalitis*.

### Anthrax

Anthrax is the second threat after smallpox that requires a major research and development effort in order to meet civilian and military needs. The most likely scenario for a bioterrorism attack is probably a covert attack, which exposes an urban population to an anthrax spore aerosol. If the release is detected or the first cases are rapidly diagnosed, rapid action can save many lives (12).

Providing the exposed population with antibiotics followed by vaccination could be lifesaving for exposed persons who would otherwise become ill with untreatable inhalation anthrax in the subsequent few weeks. Prophylactic antibiotics alone will prevent disease in persons exposed to antibiotic-susceptible organisms but incorporating vaccination into the treatment regime can greatly reduce the length of treatment with antibiotics. Without vaccination, antibiotics must be continued for 60 days; if effective vaccination can be provided, this can be reduced to 30 days (12). The current anthrax vaccine manufactured by Bioport (formerly the Michigan Department of Public Health Laboratory) is an alum-adsorbed, partially purified culture filtrate of *Bacillus anthracis* with highly protective antigen content. The schedule for administration is 0, 2, and 4 weeks and 6, 12, and 18 months. This vaccine is safe and efficacious and is being used by the armed forces to protect personnel against the use of anthrax as a weapon.

Immunization of rhesus monkeys followed by a high-dose aerosol challenge has convincingly demonstrated the capability of this vaccine to protect against aerosol challenge with *B. anthracis* spores. The multiple dose requirement, however, is a drawback for civilian use. Studies in progress may find ways to allow modification of the schedule. Vaccine supply is limited, as is production capacity. As a result, at least for the immediate future, the armed forces will require the entire available supply. This vaccine is made by a method developed before the advent of molecular biology and requires dedicated facilities because *B. anthracis* is a spore-forming organism. In addition to having a multiple-dose requirement, the vaccine is not highly purified and contains multiple extraneous proteins. The characteristics of the vaccine and the constraints on the present method of manufacturing argue strongly against procuring large amounts for civilian use when the technology and the science base exist to rapidly develop a second-generation, improved anthrax vaccine.

Anthrax depends on two toxins (lethal factor and edema factor) for virulence. A protein called protective factor is an essential component of both toxins. The protective factor content is the basis for the effectiveness of the current vaccine. A vaccine based on purified protective factor made by recombinant technology has been protective in animals. Use of a modern adjuvant with purified recombinant protective factor should make it possible to have a very effective two-dose vaccine. A recent report of the Institute of Medicine Committee on Research and Development to Improve Civilian Medical Response to Chemical and Biological Terrorism makes a strong case for a major research and development effort leading to an improved second-generation vaccine.

Questions regarding the ability of existing anthrax vaccines to protect against anthrax, strains engineered to contain additional virulence genes have been raised in Russia. Research is needed to address this and related questions about the pathogenesis of anthrax and protective immunity.

The value of vaccinating law-enforcement and emergency response personnel, who must respond to threats (real or otherwise), depends on the nature of their work and the immediacy of the threat. Laboratory personnel who must work with unknown materials and with high concentrations of known infectious materials must be vaccinated. These are additional justifications for moving ahead with a vigorous development program for anthrax and smallpox vaccines.

## Vaccine Costs and DoD Budget

Because it is recognized that some of these same BW or endemic disease agents are also potential threats to civilians, significant funds have been programmed for the Biomedical Advanced Research and Development Authority (BARDA) to stockpile vaccines against a few of the most likely pandemic disease threats or bioterrorism agents, such as pandemic influenza, anthrax, and smallpox. Although there is overlap in the missions of BARDA and DoD, their ultimate goals differ in that BARDA focuses on countermeasures for treating the population after exposure to a bioterrorism agent or in response to a pandemic, whereas the DoD aims to provide protective immunity to the armed forces prior to exposure. Today, however, while vaccination of deployed troops remains a matter of national security, the cost of vaccine development has increased to the point where, without innovation and renewed commitment, the current scope of military vaccine development efforts is not sustainable.

Protecting the health of military personnel is clearly in the best interest of the US, and vaccination is the best way to prevent endemic and BW disease threats. The question, therefore, is how to pay for the numerous vaccines that would need to be developed to accomplish this goal. One answer might be for the military to just fund all of the efforts required. Many comparisons of the cost of medical countermeasures vs. the cost of fighter jets, tanks, etc. have been made, and while it is true that the DoD medical research program is small compared with the acquisition of artillery and vehicles, such comparisons are not meaningful, as the requirement for one does not negate the requirement for the other. Realistically, the chances of major increases in the DoD budget to pay for vaccines are not good. Consequently, it will be necessary to either reduce the scope of the effort to only a few high impact diseases, or to develop novel vaccine platforms and innovative (and shortened) licensing strategies to meet the need to protect deployed troops, and for spillover benefits to the civilian community (114).

## Author Contributions

SR-K organized, researched, and wrote the main manuscript; I-KY reviewed the manuscript and was the point of contact for dengue vaccine research; RP reviewed the manuscript and was the point of contact for malaria vaccine research; J-LE reviewed the manuscript and was the point of contact for bioterrorism part; JK reviewed the manuscript and was point of contact for HIV vaccine research; RO reviewed the manuscript and had the most insights in the current vaccine military research program.

## Disclaimer

The opinions or assertions contained herein are the private views of the author, and are not to be construed as official, or as reflecting true views of the Department of the Army or the Department of Defense.

## Conflict of Interest Statement

RP is employed by the GSK group of companies and owns restricted shares of stock. GlaxoSmithKline Biologicals SA have had no role in design, interpretation, or preparation of the manuscript. The remaining authors declare that the research was conducted in the absence of any commercial or financial relationships that could be construed as a potential conflict of interest.

## Footnotes

^1^http://www.who.int/immunization/diseases/dengue/revised_SAGE_recommendations_dengue_vaccines_apr2018/en/ (Accessed: May 13, 2018).

^2^http://archive.defense.gov/pubs/ReportonBiologicalWarfareDefenseVaccineRDPrgras-July2001.pdf (Accessed: May 13, 2018).

^3^https://www.historyofvaccines.org/content/articles/biological-weapons-bioterrorism-and-vaccines (Accessed: May 13, 2018).

## References

[1] Dye C. After 2015: infectious diseases in a new era of health and development. Philos Trans R Soc Lond B Biol Sci (2014) 369(1645):20130426. 10.1098/rstb.2013.0426 24821913 PMC4024220

[2] WHO. The Top 10 Causes of Death. World Health Organization (2017). Available from: http://www.who.int/mediacentre/factsheets/fs310/en/ (Accessed: May 13, 2018).

[3] Gordon JE General consideration of modes of transmission. Medical Department USA. Preventive Medicine in World War II: Communicable Diseases Transmitted Chiefly Through Respiratory and Alimentary Tracts. Washington, DC: Office of the Surgeon General, Department of the Army (1958). Available from: http://history.amedd.army.mil/booksdocs/wwii/PM4/CH1.htm (Accessed: May 13, 2018).

[4] Committee on a Strategy for Minimizing the Impact of Naturally Occurring Infectious Diseases of Military Importance: Vaccine Issues in the U.S. Military Lemon SM Thaul S Fisseha S O’Maonaigh HC, editors. Protecting Our Forces: Improving Vaccine Acquisition and Availability in the U.S. Military. Washington, DC: National Academies Press (2002). 158 p. Available from: http://www.nap.edu/catalog/10483.html 25057623

[5] Stanhope B-J The Evolution of Preventive Medicine in the United States Army, 1607–1939. Anderson RS, editor. Washington, DC: Office of the Surgeon General, Department of the Army Available from: http://history.amedd.army.mil/booksdocs/wwii/Malaria/chapterI.htm

[6] Grabenstein JD Pittman PR Greenwood JT Engler RJ. Immunization to protect the US Armed Forces: heritage, current practice, and prospects. Epidemiol Rev (2006) 28:3–26. 10.1093/epirev/mxj003 16763072

[7] Artenstein AW. Vaccines for military use. Vaccine (2009) 27(Suppl 4):D16–22. 10.1016/j.vaccine.2009.07.044 19837279

[8] The National Security Strategies of the United States of America. (2002). Available from: https://www.state.gov/documents/organization/63562.pdf.

[9] Gibbons RV Nisalak A Yoon IK Tannitisupawong D Rungsimunpaiboon K Vaughn DW A model international partnership for community-based research on vaccine-preventable diseases: the Kamphaeng Phet-AFRIMS Virology Research Unit (KAVRU). Vaccine (2013) 31(41):4487–500. 10.1016/j.vaccine.2013.07.082 23933334

[10] Tschanz DW Smallpox and the American revolution. Command: Military History, Strategy & Analysis (1995) (32):33.

[11] Christenson S Lackland Gets a Shot at Smallpox Vaccine for the First Time in a Decade, 14 Air Force Volunteers in San Antonio Are Inoculated Against a Disease the World Once Feared. San Antonio, TX: San Antonio Express-News (2003).

[12] Russell PK Vaccines in civilian defense against bioterrorism. Emerg Infect Dis (1999) 5(4):531–3. 10.3201/eid0504.990413 10458959 PMC2627741

[13] Golden JW Hooper JW. The strategic use of novel smallpox vaccines in the post-eradication world. Expert Rev Vaccines (2011) 10(7):1021–35. 10.1586/erv.11.46 21806397 PMC9491137

[14] Poland GA Grabenstein JD Neff JM. The US smallpox vaccination program: a review of a large modern era smallpox vaccination implementation program. Vaccine (2005) 23(17–18):2078–81. 10.1016/j.vaccine.2005.01.012 15755574

[15] Artenstein AW Grabenstein JD. Smallpox vaccines for biodefense: need and feasibility. Expert Rev Vaccines (2008) 7(8):1225–37. 10.1586/14760584.7.8.1225 18844596 PMC9709930

[16] Petersen BW Harms TJ Reynolds MG Harrison LH. Use of vaccinia virus smallpox vaccine in laboratory and health care personnel at risk for occupational exposure to orthopoxviruses – recommendations of the Advisory Committee on Immunization Practices (ACIP), 2015. MMWR Morb Mortal Wkly Rep (2016) 65(10):257–62. 10.15585/mmwr.mm6510a2 26985679

[17] Barnett ED Yellow fever: epidemiology and prevention. Clin Infect Dis (2007) 44(6):850–6. 10.1086/511869 17304460

[18] Kitchen LW Vaughn DW. Role of U.S. military research programs in the development of U.S.-licensed vaccines for naturally occurring infectious diseases. Vaccine (2007) 25(41):7017–30. 10.1016/j.vaccine.2007.07.030 17728025

[19] Gershman MD Angelo KM Ritchey J Greenberg DP Muhammad RD Brunette G Addressing a yellow fever vaccine shortage – United States, 2016–2017. MMWR Morb Mortal Wkly Rep (2017) 66(17):457–9. 10.15585/mmwr.mm6617e2 28472025 PMC5687078

[20] Roukens AH Vossen AC Bredenbeek PJ van Dissel JT Visser LG. Intradermally administered yellow fever vaccine at reduced dose induces a protective immune response: a randomized controlled non-inferiority trial. PLoS One (2008) 3(4):e1993. 10.1371/journal.pone.0001993 18431480 PMC2297511

[21] Ahuka-Mundeke S Casey RM Harris JB Dixon MG Nsele PM Kizito GM Immunogenicity of fractional-dose vaccine during a yellow fever outbreak – preliminary report. N Engl J Med (2018). 10.1056/NEJMoa1710430 PMC7064153 29443626

[22] Steele AD Hay Burgess DC Diaz Z Carey ME Zaidi AK. Challenges and opportunities for typhoid fever control: a call for coordinated action. Clin Infect Dis (2016) 62(Suppl 1):S4–8. 10.1093/cid/civ976 26933019 PMC4772836

[23] Watson DA Musher DM Jacobson JW Verhoef J. A brief history of the pneumococcus in biomedical research: a panoply of scientific discovery. Clin Infect Dis (1993) 17(5):913–24. 10.1093/clinids/17.5.913 8286641

[24] Bonten MJ Huijts SM Bolkenbaas M Webber C Patterson S Gault S Polysaccharide conjugate vaccine against pneumococcal pneumonia in adults. N Engl J Med (2015) 372(12):1114–25. 10.1056/NEJMoa1408544 25785969

[25] Pilishvili T Bennett NM. Pneumococcal disease prevention among adults: strategies for the use of pneumococcal vaccines. Am J Prev Med (2015) 49(6 Suppl 4):S383–90. 10.1016/j.amepre.2015.09.008 26590438

[26] Moore MR Link-Gelles R Schaffner W Lynfield R Holtzman C Harrison LH Effectiveness of 13-valent pneumococcal conjugate vaccine for prevention of invasive pneumococcal disease in children in the USA: a matched case-control study. Lancet Respir Med (2016) 4(5):399–406. 10.1016/S2213-2600(16)00052-7 26987984

[27] Ottolini MG Burnett MW. History of U.S. military contributions to the study of respiratory infections. Mil Med (2005) 170(4 Suppl):66–70. 10.7205/MILMED.170.4S.66 15916285

[28] Meiklejohn G Eickhoff TC Graves P. Antibody response of young adults to experimental influenza A/New Jersey/76 virus vaccines. J Infect Dis (1977) 136(Suppl):S456–9. 10.1093/infdis/136.Supplement_3.S456 606766

[29] Meiklejohn G Eickhoff TC Graves P Josephine I Antigenic drift and efficacy of influenza virus vaccines, 1976–1977. J Infect Dis (1978) 138(5):618–24. 10.1093/infdis/138.5.618 712117

[30] Hoke CH Jr Hopkins JA Meiklejohn G Mostow SR. Comparison of several wild-type influenza viruses in the ferret tracheal organ culture system. Rev Infect Dis (1979) 1(6):946–54. 10.1093/clinids/1.6.946 399387

[31] Gremillion DH Meiklejohn G Graves P Josephine I Efficacy of single-dose influenza in Air Force recruits. J Infect Dis (1983) 147(6):1099 10.1093/infdis/147.6.1099 6854064

[32] Radin JM Hawksworth AW Blair PJ Faix DJ Raman R Russell KL Dramatic decline of respiratory illness among US military recruits after the renewed use of adenovirus vaccines. Clin Infect Dis (2014) 59(7):962–8. 10.1093/cid/ciu507 24991024

[33] Artenstein AW Opal JM Opal SM Tramont EC Peter G Russell PK. History of U.S. military contributions to the study of vaccines against infectious diseases. Mil Med (2005) 170(4 Suppl):3–11. 10.7205/MILMED.170.4S.3 15916278

[34] Plotkin SA. The history of rubella and rubella vaccination leading to elimination. Clin Infect Dis (2006) 43(Suppl 3):S164–8. 10.1086/505950 16998777

[35] Watson JC Hadler SC Dykewicz CA Reef S Phillips L Measles, mumps, and rubella – vaccine use and strategies for elimination of measles, rubella, and congenital rubella syndrome and control of mumps: recommendations of the Advisory Committee on Immunization Practices (ACIP). MMWR Recomm Rep (1998) 47(RR–8):1–57. 9639369

[36] Paulke-Korinek M Kollaritsch H. Japanese encephalitis and vaccines: past and future prospects. Wien Klin Wochenschr (2008) 120(19–20 Suppl 4):15–9. 10.1007/s00508-008-1071-9 19066766

[37] Jelinek T. IXIARO updated: overview of clinical trials and developments with the inactivated vaccine against Japanese encephalitis. Expert Rev Vaccines (2013) 12(8):859–69. 10.1586/14760584.2013.835638 23984958

[38] Erra EO Kantele A. The Vero cell-derived, inactivated, SA14-14-2 strain-based vaccine (Ixiaro) for prevention of Japanese encephalitis. Expert Rev Vaccines (2015) 14(9):1167–79. 10.1586/14760584.2015.1061939 26162529

[39] Appaiahgari MB Vrati S IMOJEV((R)): a yellow fever virus-based novel Japanese encephalitis vaccine. Expert Rev Vaccines (2010) 9(12):1371–84. 10.1586/erv.10.139 21105774

[40] Goldschneider I Gotschlich EC Artenstein MS Human immunity to the meningococcus. I. The role of humoral antibodies. J Exp Med (1969) 129(6):1307–26. 10.1084/jem.129.6.1307 4977280 PMC2138650

[41] Goldschneider I Gotschlich EC Artenstein MS Human immunity to the meningococcus. II. Development of natural immunity. J Exp Med (1969) 129(6):1327–48. 10.1084/jem.129.6.1327 4977281 PMC2138665

[42] Gotschlich EC Goldschneider I Artenstein MS Human immunity to the meningococcus. IV. Immunogenicity of group A and group C meningococcal polysaccharides in human volunteers. J Exp Med (1969) 129(6):1367–84. 10.1084/jem.129.6.1367 4977283 PMC2138657

[43] Gotschlich EC Goldschneider I Artenstein MS Human immunity to the meningococcus. V. The effect of immunization with meningococcal group C polysaccharide on the carrier state. J Exp Med (1969) 129(6):1385–95. 10.1084/jem.129.6.1385 4977284 PMC2138662

[44] Findlow J. Meningococcal group B vaccines. Hum Vaccin Immunother (2013) 9(6):1387–8. 10.4161/hv.24689 23732894 PMC3901838

[45] Leca M Bornet C Montana M Curti C Vanelle P Meningococcal vaccines: current state and future outlook. Pathol Biol (Paris) (2015) 63(3):144–51. 10.1016/j.patbio.2015.04.003 25986879

[46] Honigsbaum M The Fever Trail: In Search of the Cure for Malaria. New York: Farrar, Straus and Giroux (2002).

[47] Ockenhouse CF Magill A Smith D Milhous W. History of U.S. military contributions to the study of malaria. Mil Med (2005) 170(4 Suppl):12–6. 10.7205/MILMED.170.4S.12 15916279

[48] Masterson KM The Malaria Project. New York: Penguin (2014).

[49] Young MD Moore DV Chloroquine resistance in *Plasmodium falciparum*. Am J Trop Med Hyg (1961) 10:317–20. 10.4269/ajtmh.1961.10.317 13787478

[50] Tigertt WD The army malaria research program. Ann Intern Med (1969) 70(1):150–3. 10.7326/0003-4819-70-1-150 4974174

[51] Rieckmann KH. Human immunization with attenuated sporozoites. Bull World Health Organ (1990) 68(Suppl):13–6. 2094578 PMC2393053

[52] Hoffman SL Goh LM Luke TC Schneider I Le TP Doolan DL Protection of humans against malaria by immunization with radiation-attenuated *Plasmodium falciparum* sporozoites. J Infect Dis (2002) 185(8):1155–64. 10.1086/339409 11930326

[53] Seder RA Chang LJ Enama ME Zephir KL Sarwar UN Gordon IJ Protection against malaria by intravenous immunization with a nonreplicating sporozoite vaccine. Science (2013) 341(6152):1359–65. 10.1126/science.1241800 23929949

[54] Ishizuka AS Lyke KE DeZure A Berry AA Richie TL Mendoza FH Protection against malaria at 1 year and immune correlates following PfSPZ vaccination. Nat Med (2016) 22(6):614–23. 10.1038/nm.4110 27158907 PMC11294733

[55] Sissoko MS Healy SA Katile A Omaswa F Zaidi I Gabriel EE Safety and efficacy of PfSPZ vaccine against *Plasmodium falciparum* via direct venous inoculation in healthy malaria-exposed adults in Mali: a randomised, double-blind phase 1 trial. Lancet Infect Dis (2017) 17(5):498–509. 10.1016/S1473-3099(17)30104-4 28216244 PMC6803168

[56] Hoffman SL Vekemans J Richie TL Duffy PE. The March toward malaria vaccines. Am J Prev Med (2015) 49(6 Suppl 4):S319–33. 10.1016/j.amepre.2015.09.011 26590432 PMC5077160

[57] Ellis J Ozaki LS Gwadz RW Cochrane AH Nussenzweig V Nussenzweig RS Cloning and expression in *E. coli* of the malarial sporozoite surface antigen gene from *Plasmodium knowlesi*. Nature (1983) 302(5908):536–8. 10.1038/302536a0 6339949

[58] Hoffman SL Wistar R Jr Ballou WR Hollingdale MR Wirtz RA Schneider I Immunity to malaria and naturally acquired antibodies to the circumsporozoite protein of *Plasmodium falciparum*. N Engl J Med (1986) 315(10):601–6. 10.1056/NEJM198609043151001 3526148

[59] Good MF Pombo D Quakyi IA Riley EM Houghten RA Menon A Human T-cell recognition of the circumsporozoite protein of *Plasmodium falciparum*: immunodominant T-cell domains map to the polymorphic regions of the molecule. Proc Natl Acad Sci U S A (1988) 85(4):1199–203. 10.1073/pnas.85.4.1199 2448793 PMC279734

[60] Stoute JA Slaoui M Heppner DG Momin P Kester KE Desmons P A preliminary evaluation of a recombinant circumsporozoite protein vaccine against *Plasmodium falciparum* malaria. RTS,S Malaria Vaccine Evaluation Group. N Engl J Med (1997) 336(2):86–91. 10.1056/NEJM199701093360202 8988885

[61] Gordon DM McGovern TW Krzych U Cohen JC Schneider I LaChance R Safety, immunogenicity, and efficacy of a recombinantly produced *Plasmodium falciparum* circumsporozoite protein-hepatitis B surface antigen subunit vaccine. J Infect Dis (1995) 171(6):1576–85. 10.1093/infdis/171.6.1576 7769295

[62] Rts SCTP Agnandji ST Lell B Soulanoudjingar SS Fernandes JF Abossolo BP First results of phase 3 trial of RTS,S/AS01 malaria vaccine in African children. N Engl J Med (2011) 365(20):1863–75. 10.1056/NEJMoa1102287 22007715

[63] Rts SCTP Agnandji ST Lell B Fernandes JF Abossolo BP Methogo BG A phase 3 trial of RTS,S/AS01 malaria vaccine in African infants. N Engl J Med (2012) 367(24):2284–95. 10.1056/NEJMoa1208394 23136909 PMC10915853

[64] Olotu A Fegan G Wambua J Nyangweso G Awuondo KO Leach A Four-year efficacy of RTS,S/AS01E and its interaction with malaria exposure. N Engl J Med (2013) 368(12):1111–20. 10.1056/NEJMoa1207564 23514288 PMC5156295

[65] WHO. Malaria vaccine: WHO position paper 2016. Weekly Epidemiological Record. Geneva: WHO (2016) p. 33–52. Available from: http://www.who.int/wer

[66] Freedman DO Clinical practice. Malaria prevention in short-term travelers. N Engl J Med (2008) 359(6):603–12. 10.1056/NEJMcp0803572 18687641

[67] Regules JA Cicatelli SB Bennett JW Paolino KM Twomey PS Moon JE Fractional third and fourth dose of RTS,S/AS01 malaria candidate vaccine: a phase 2a controlled human malaria parasite infection and immunogenicity study. J Infect Dis (2016) 214(5):762–71. 10.1093/infdis/jiw237 27296848

[68] Birkett AJ. Status of vaccine research and development of vaccines for malaria. Vaccine (2016) 34(26):2915–20. 10.1016/j.vaccine.2015.12.074 26993333

[69] Gibbons RV Streitz M Babina T Fried JR. Dengue and US military operations from the Spanish-American War through today. Emerg Infect Dis (2012) 18(4):623–30. 10.3201/eid1804.110134 22469290 PMC3309667

[70] Monath TP. Dengue: the risk to developed and developing countries. Proc Natl Acad Sci U S A (1994) 91(7):2395–400. 10.1073/pnas.91.7.2395 8146129 PMC43378

[71] Simmons CP McPherson K Van Vinh Chau N Hoai Tam DT Young P Mackenzie J Recent advances in dengue pathogenesis and clinical management. Vaccine (2015) 33(50):7061–8. 10.1016/j.vaccine.2015.09.103 26458808

[72] Thomas SJ Endy TP. Current issues in dengue vaccination. Curr Opin Infect Dis (2013) 26(5):429–34. 10.1097/01.qco.0000433310.28771.cc 23963259

[73] Thomas SJ Endy TP. Vaccines for the prevention of dengue: development update. Hum Vaccin (2011) 7(6):674–84. 10.4161/hv.7.6.14985 21508679

[74] Guy B Barrere B Malinowski C Saville M Teyssou R Lang J. From research to phase III: preclinical, industrial and clinical development of the Sanofi Pasteur tetravalent dengue vaccine. Vaccine (2011) 29(42):7229–41. 10.1016/j.vaccine.2011.06.094 21745521

[75] Sabchareon A Wallace D Sirivichayakul C Limkittikul K Chanthavanich P Suvannadabba S Protective efficacy of the recombinant, live-attenuated, CYD tetravalent dengue vaccine in Thai schoolchildren: a randomised, controlled phase 2b trial. Lancet (2012) 380(9853):1559–67. 10.1016/S0140-6736(12)61428-7 22975340

[76] Capeding MR Tran NH Hadinegoro SR Ismail HI Chotpitayasunondh T Chua MN Clinical efficacy and safety of a novel tetravalent dengue vaccine in healthy children in Asia: a phase 3, randomised, observer-masked, placebo-controlled trial. Lancet (2014) 384(9951):1358–65. 10.1016/S0140-6736(14)61060-6 25018116

[77] Villar L Dayan GH Arredondo-Garcia JL Rivera DM Cunha R Deseda C Efficacy of a tetravalent dengue vaccine in children in Latin America. N Engl J Med (2015) 372(2):113–23. 10.1056/NEJMoa1411037 25365753

[78] WHO. Dengue vaccine: WHO position paper 2016. Weekly Epidemiological Record. Geneva: WHO (2016). p. 349–64. Available from: http://www.who.int/wer

[79] Bauer K Esquilin IO Cornier AS Thomas SJ Quintero Del Rio AI Bertran-Pasarell J A phase II, randomized, safety and immunogenicity trial of a re-derived, live-attenuated dengue virus vaccine in healthy children and adults living in Puerto Rico. Am J Trop Med Hyg (2015) 93(3):441–53. 10.4269/ajtmh.14-0625 26175027 PMC4559678

[80] Schmidt AC Lin L Martinez LJ Ruck RC Eckels KH Collard A Phase 1 Randomized Study of a Tetravalent Dengue Purified Inactivated Vaccine in Healthy Adults in the United States. Am J Trop Med Hyg (2017) 96(6):1325–37. 10.4269/ajtmh.16-0634 28719287 PMC5462566

[81] Diaz C Lin L Martinez LJ Eckels KH Campos M Jarman RG Phase 1 Randomized Study of a Tetravalent Dengue Purified Inactivated Vaccine in Healthy Adults from Puerto Rico. Am J Trop Med Hyg (2018) 98(5):1435–43. 10.4269/ajtmh.17-0627 29512481 PMC5953365

[82] Danko JR Kochel T Teneza-Mora N Luke TC Raviprakash K Sun P Safety and immunogenicity of a tetravalent dengue DNA vaccine administered with a cationic lipid-based adjuvant in a phase 1 clinical trial. Am J Trop Med Hyg (2018) 98(3):849–56. 10.4269/ajtmh.17-0416 29363446 PMC5930886

[83] Reilly L. U.S. Military HIV Research Program: successfully integrating HIV vaccine research with prevention, care, and treatment. Mil Med (2010) 175(7 Suppl):42–4. 10.7205/MILMED-D-10-00168 23634478

[84] Brown AE Nitayaphan S Foundations for a phase III human immunodeficiency virus vaccine trial: a decade of Thai-U.S. Army collaborative research. Mil Med (2004) 169(8):588–93. 15379068 10.7205/milmed.169.8.588

[85] Viputtijul K de Souza M Trichavaroj R Carr JK Tovanabutra S McCutchan FE Heterosexually acquired CRF01_AE/B recombinant HIV type 1 found in Thailand. AIDS Res Hum Retroviruses (2002) 18(16):1235–7. 10.1089/08892220260387986 12494923

[86] Mason CJ Markowitz LE Kitsiripornchai S Jugsudee A Sirisopana N Torugsa K Declining prevalence of HIV-1 infection in young Thai men. AIDS (1995) 9(9):1061–5. 10.1097/00002030-199509000-00012 8527079

[87] Nitayaphan S Khamboonruang C Sirisophana N Morgan P Chiu J Duliege AM A phase I/II trial of HIV SF2 gp120/MF59 vaccine in seronegative thais.AFRIMS-RIHES Vaccine Evaluation Group. Armed Forces Research Institute of Medical Sciences and the Research Institute for Health Sciences. Vaccine (2000) 18(15):1448–55. 10.1016/S0264-410X(99)00421-1 10618542

[88] Pitisuttithum P Nitayaphan S Thongcharoen P Khamboonruang C Kim J de Souza M Safety and immunogenicity of combinations of recombinant subtype E and B human immunodeficiency virus type 1 envelope glycoprotein 120 vaccines in healthy Thai adults. J Infect Dis (2003) 188(2):219–27. 10.1086/376506 12854076

[89] Evans TG Keefer MC Weinhold KJ Wolff M Montefiori D Gorse GJ A canarypox vaccine expressing multiple human immunodeficiency virus type 1 genes given alone or with rgp120 elicits broad and durable CD8+ cytotoxic T lymphocyte responses in seronegative volunteers. J Infect Dis (1999) 180(2):290–8. 10.1086/314895 10395842

[90] Belshe RB Stevens C Gorse GJ Buchbinder S Weinhold K Sheppard H Safety and immunogenicity of a canarypox-vectored human immunodeficiency virus type 1 vaccine with or without gp120: a phase 2 study in higher- and lower-risk volunteers. J Infect Dis (2001) 183(9):1343–52. 10.1086/319863 11294665

[91] Gupta K Hudgens M Corey L McElrath MJ Weinhold K Montefiori DC Safety and immunogenicity of a high-titered canarypox vaccine in combination with rgp120 in a diverse population of HIV-1-uninfected adults: AIDS Vaccine Evaluation Group Protocol 022A. J Acquir Immune Defic Syndr (2002) 29(3):254–61. 10.1097/00042560-200203010-00005 11873074

[92] Nitayaphan S Pitisuttithum P Karnasuta C Eamsila C de Souza M Morgan P Safety and immunogenicity of an HIV subtype B and E prime-boost vaccine combination in HIV-negative Thai adults. J Infect Dis (2004) 190(4):702–6. 10.1086/422258 15272397

[93] Thongcharoen P Suriyanon V Paris RM Khamboonruang C de Souza MS Ratto-Kim S A phase 1/2 comparative vaccine trial of the safety and immunogenicity of a CRF01_AE (subtype E) candidate vaccine: ALVAC-HIV (vCP1521) prime with oligomeric gp160 (92TH023/LAI-DID) or bivalent gp120 (CM235/SF2) boost. J Acquir Immune Defic Syndr (2007) 46(1):48–55. 10.1097/QAI.0b013e3181354bd7 17909315

[94] Rerks-Ngarm S Pitisuttithum P Nitayaphan S Kaewkungwal J Chiu J Paris R Vaccination with ALVAC and AIDSVAX to prevent HIV-1 infection in Thailand. N Engl J Med (2009) 361(23):2209–20. 10.1056/NEJMoa0908492 19843557

[95] Currier JR Ngauy V de Souza MS Ratto-Kim S Cox JH Polonis VR Phase I safety and immunogenicity evaluation of MVA-CMDR, a multigenic, recombinant modified vaccinia Ankara-HIV-1 vaccine candidate. PLoS One (2010) 5(11):e13983. 10.1371/journal.pone.0013983 21085591 PMC2981570

[96] Ake JA Schuetz A Pegu P Wieczorek L Eller MA Kibuuka H Safety and immunogenicity of PENNVAX-G DNA prime administered by biojector 2000 or CELLECTRA electroporation device with modified vaccinia ankara-CMDR boost. J Infect Dis (2017) 216(9):1080–90. 10.1093/infdis/jix456 28968759 PMC5853809

[97] Haynes BF Gilbert PB McElrath MJ Zolla-Pazner S Tomaras GD Alam SM Immune-correlates analysis of an HIV-1 vaccine efficacy trial. N Engl J Med (2012) 366(14):1275–86. 10.1056/NEJMoa1113425 22475592 PMC3371689

[98] Tomaras GD Plotkin SA. Complex immune correlates of protection in HIV-1 vaccine efficacy trials. Immunol Rev (2017) 275(1):245–61. 10.1111/imr.12514 28133811 PMC5330182

[99] Thornton SA Sherman SS Farkas T Zhong W Torres P Jiang X. Gastroenteritis in US marines during operation Iraqi freedom. Clin Infect Dis (2005) 40(4):519–25. 10.1086/427501 15712073

[100] DeFraites RF Sanchez JL Brandt CA Kadlec RP Haberberger RL Lin JJ An outbreak of *Campylobacter* enteritis associated with a community water supply on a U.S. military installation. MSMR (2014) 21(11):10–5. 25436877

[101] Riddle MS Tribble DR Cachafiero SP Putnam SD Hooper TI. Development of a travelers’ diarrhea vaccine for the military: how much is an ounce of prevention really worth? Vaccine (2008) 26(20):2490–502. 10.1016/j.vaccine.2008.03.008 18417259

[102] Tallant A Porter CK Putnam SD Tribble DR Hooper TI Riddle MS. Relative cost-effectiveness of a norovirus vaccine in the deployed military setting compared to a vaccine against *Campylobacter* sp., ETEC, and *Shigella* sp. Vaccine (2014) 32(40):5156–62. 10.1016/j.vaccine.2014.07.070 25086264

[103] Walker RI. An assessment of enterotoxigenic *Escherichia coli* and *Shigella* vaccine candidates for infants and children. Vaccine (2015) 33(8):954–65. 10.1016/j.vaccine.2014.11.049 25482842

[104] Kelly DJ Richards AL Temenak J Strickman D Dasch GA. The past and present threat of rickettsial diseases to military medicine and international public health. Clin Infect Dis (2002) 34(Suppl 4):S145–69. 10.1086/339908 12016590

[105] Hoke CH Jr Pace-Templeton J Pittman P Malinoski FJ Gibbs P Ulderich T US Military contributions to the global response to pandemic chikungunya. Vaccine (2012) 30(47):6713–20. 10.1016/j.vaccine.2012.08.025 22940380

[106] Smalley C Erasmus JH Chesson CB Beasley DWC. Status of research and development of vaccines for chikungunya. Vaccine (2016) 34(26):2976–81. 10.1016/j.vaccine.2016.03.076 27026149

[107] Morens DM Fauci AS Pandemic Zika: a formidable challenge to medicine and public health. J Infect Dis (2017) 216(Suppl_10):S857–9. 10.1093/infdis/jix383 29267908 PMC5853239

[108] Gubler DJ Vasilakis N Musso D. History and emergence of Zika virus. J Infect Dis (2017) 216(Suppl_10):S860–7. 10.1093/infdis/jix451 29267917 PMC5853376

[109] Morabito KM Graham BS. Zika virus vaccine development. J Infect Dis (2017) 216(Suppl_10):S957–63. 10.1093/infdis/jix464 29267918 PMC5854011

[110] Ferguson NM Cucunuba ZM Dorigatti I Nedjati-Gilani GL Donnelly CA Basanez MG Epidemiology. Countering the Zika epidemic in Latin America. Science (2016) 353(6297):353–4. 10.1126/science.aag0219 27417493 PMC5475255

[111] Schmaljohn CS Vaccines for hantaviruses: progress and issues. Expert Rev Vaccines (2012) 11(5):511–3. 10.1586/erv.12.15 22827236

[112] McClain DJ Summers PL Harrison SA Schmaljohn AL Schmaljohn CS. Clinical evaluation of a vaccinia-vectored Hantaan virus vaccine. J Med Virol (2000) 60(1):77–85. 10.1002/(SICI)1096-9071(200001)60:1<77::AID-JMV13>3.0.CO;2-S 10568767

[113] Boudreau EF Josleyn M Ullman D Fisher D Dalrymple L Sellers-Myers K A phase 1 clinical trial of Hantaan virus and Puumala virus M-segment DNA vaccines for hemorrhagic fever with renal syndrome. Vaccine (2012) 30(11):1951–8. 10.1016/j.vaccine.2012.01.024 22248821

[114] Schmaljohn CS Smith LA Friedlander AM. Military vaccines in today’s environment. Hum Vaccin Immunother (2012) 8(8):1126–8. 10.4161/hv.20503 22854669 PMC3551885

[115] Riedel S Biological warfare and bioterrorism: a historical review. Proc (Bayl Univ Med Cent) (2004) 17(4):400–6. 10.1080/08998280.2004.11928002 16200127 PMC1200679

[116] Lutwick LI Nierengarten MB. Vaccines for category A bioterrorism diseases. Expert Opin Biol Ther (2002) 2(8):883–93. 10.1517/14712598.2.8.883 12517267

[117] Kimmel SR Mahoney MC Zimmerman RK. Vaccines and bioterrorism: smallpox and anthrax. J Fam Pract (2003) 52(1 Suppl):S56–61. 12556279

